# Single-incision plus one-port laparoscopy surgery versus conventional multi-port laparoscopy surgery for colorectal cancer: a systematic review and meta-analysis

**DOI:** 10.1007/s00384-024-04630-x

**Published:** 2024-04-29

**Authors:** Jian Kong, Meng-qi Wu, Shuai Yan, Zheng-fei Zhao, Hui Yao

**Affiliations:** 1 Department of General Surgery(Gastrointestinal Surgery), The Affiliated Hospital of Southwest Medical University, Sichuan Province Luzhou, China; 2 Southwest Medical University, Sichuan Province Luzhou, China

**Keywords:** Single-incision plus one-port, SILS + 1, CLS, Laparoscopy surgery, Meta-analysis

## Abstract

**Objective:**

The efficacy of single-incision plus one-port laparoscopic surgery (SILS + 1) versus conventional laparoscopic surgery (CLS) for colorectal cancer treatment remains unclear. This study compares the short-term and long-term outcomes of SILS + 1 and CLS using a high-quality systematic review and meta-analysis.

**Method:**

Literature search followed the Preferred Reporting Items for Systematic Reviews and Meta-Analyses (PRISMA) guidelines, drawing from PubMed, Embase, Web of Science, and the Cochrane Library until December 10, 2023. Statistical analysis was conducted using RevMan and Stata.

**Result:**

The review and meta-analysis included seven studies with 1740 colorectal cancer patients. Compared to CLS, SILS + 1 showed significant improvements in operation time (WMD =  − 18.33, *P* < 0.00001), blood loss (WMD =  − 21.31, *P* < 0.00001), incision length (WMD =  − 2.07, *P* < 0.00001), time to first defecation (WMD =  − 14.91, *P* = 0.009), time to oral intake (WMD =  − 11.46, *P* = 0.04), and time to ambulation (WMD =  − 11.52, *P* = 0.01). There were no significant differences in lymph node harvest, resection margins, complications, anastomotic leakage, hospital stay, disease-free survival, overall survival, and postoperative recurrence.

**Conclusions:**

Compared to CLS, SILS + 1 demonstrates superiority in shortening the surgical incision and promoting postoperative recovery. SILS + 1 can provide a safe and feasible alternative to CLS.

**Supplementary Information:**

The online version contains supplementary material available at 10.1007/s00384-024-04630-x.

## Introduction

Since the 1990s, laparoscopic technology has been widely adopted and has replaced open surgery as a superior choice for colorectal cancer surgery [1–3]. Laparoscopic surgery not only maintains good oncological treatment outcomes but also offers superior aesthetics, reduces postoperative pain, accelerates postoperative recovery, shortens hospital stays, and decreases the incidence of perioperative complications [4]. Such technological progress has directed the development of colorectal cancer surgery and is driving the advancement of laparoscopic surgery towards further minimally invasive surgery.

The single-incision laparoscopic surgery (SILS) is considered typical of minimally invasive surgical advancement, which has been reported by numerous studies in the last decade [5–7]. Single-incision laparoscopic colorectal surgery typically involves a single incision around the umbilicus as the surgical access route, through which the surgical specimen is extracted. This approach offers advantages such as reduced incision length, decreased pain, improved cosmetic outcomes, and enhanced postoperative recovery [8–11]. However, its widespread adoption is hindered by the increased technical challenges posed to surgeons. These challenges may be attributed to various complexities in the SILS procedure, including collision of device movements, suboptimal surgical site exposure, and real-time visualization [12, 14], and cause an increased risk of intraoperative vascular and tissue damage [15].

To address these technical challenges, an additional port was introduced in the SILS, known as single-incision plus one-port laparoscopy (SILS + 1) [16]. Supplementary ports restore the triangulation of laparoscopic surgery, overcoming the aforementioned challenges, while striving to preserve as many advantages of SILS as possible [17, 18]. As surgery has evolved from five-port laparoscopy to more minimally invasive techniques, SILS + 1 is considered a critical learning stage. Therefore, SILS + 1 deserves further attention in the current technological realm.

However, there is currently a lack of evidence-based substantiation regarding the safety and reliability of SILS + 1 technology. Recent clinical research outcomes are limited by small sample sizes and inadequate quality of evidence [16, 17]. Therefore, the objective of this meta-analysis is to provide a comprehensive assessment of the short-term and medium-to-long-term efficacy comparisons between SILS + 1 and conventional laparoscopic surgery (CLS) in patients with colorectal cancer.

## Methods

### Search strategy

This meta-analysis adhered to the PRISMA guidelines [19] (Preferred Reporting Items for Systematic Reviews and Meta-Analyses) (see Supplementary Table 1) and systematically gathered pertinent information on SILS + 1 and CLS from PubMed, Embase, Web of Science, and the Cochrane Library databases. The systematic review protocol has been duly registered on PROSPERO (CRD 42023492696). The search strategy encompassed the following terms: (1) “single-incision plus one port” OR “single-incision plus one-port” OR “SILS + 1” OR “two port” OR “two Incision” OR “reduced-port” OR “RPLS” OR “PRS”; (2) “multi* port*” OR “multi* incision*” OR standard OR traditional OR conventional; (3) colon* OR colorectal OR rectal OR rectum; and (4) cancer* OR tumour* OR neoplasm* OR malignant*. The search was concluded on December 10, 2023.

### Exclusion and inclusion criteria

Excluded from consideration were studies involving three-port or four-port laparoscopic surgeries, as well as reduced-port laparoscopic surgeries lacking specific surgical details or robot-assisted laparoscopic surgeries. Additionally, studies implementing the enhanced recovery after surgery (ERAS) protocol were also excluded, as this approach has been proven to significantly promote postoperative recovery in patients [20]. Articles not published in English and those inaccessible for full-text retrieval were also excluded. Content such as reviews, letters, editorials, case reports, animal experimental studies, and conference abstracts did not meet the inclusion criteria.

Included were only comparative studies featuring at least one assessable primary or secondary outcome. In instances of multiple redundant studies, preference was given to the most recent or comprehensive reports. Initially, all identified titles and abstracts underwent independent review by two evaluators (Kong and Wu). Subsequently, these two reviewers independently examined the full texts of potentially relevant articles. In the event of discrepancies, a third reviewer (Zhao) was consulted, and the relevant terms were discussed until a consensus was reached.

### Data extraction and quality assessment

From all the studies included in the analysis, the following relevant information was extracted: reference, country/region, sample size, age, gender (male), BMI, tumor grade, and study design. Primary outcomes comprised operating time, blood loss, and complications. Secondary outcomes included incision length, the number of harvested lymph nodes, proximal and distal resection margins, time to first flatus and defecation, time to first oral intake and ambulation, hospital stay, anastomotic leakage, recurrence, overall survival rate, and disease-free survival rate. Data required for survival analysis were extracted using Engauge Digitizer (version 11.1) and tables provided by Tierney et al. [21], allowing conversion to the necessary data effect size.

The assessment of the quality of randomized controlled trials (RCTs) employed the Cochrane risk-of-bias tool [22], while cohort studies were evaluated using the Newcastle–Ottawa Scale (NOS) [23]. RCTs with a minimum score of 4 and cohort studies with a minimum score of 7 were considered to have higher methodological quality. Studies with low quality were excluded from the analysis.

### Statistical analysis

Continuous variables and dichotomous variables underwent analysis using weighted mean differences (WMD) and odds ratios (OR), respectively. Heterogeneity among studies was assessed using *τ*^2^ and *I*^2^, where *I*^2^ ≤ 50% indicated low heterogeneity, and a fixed-effects model was used; *I*^2^ > 50% indicated high heterogeneity, and a random-effects model was applied. *I*^2^ ≥ 75% was considered significant heterogeneity. In the presence of significant heterogeneity, methods such as meta-regression and subgroup analysis were used to explore the sources of heterogeneity; otherwise, descriptive analysis was performed. Sensitivity analysis was conducted to examine the impact of each study on the overall results, ensuring stability and reliability. Quantitative analysis of publication bias was performed using the Harbord test and Egger test. A significance level of *P* < 0.05 indicated significant publication bias. Statistical analyses were performed using RevMan 5.4 (The Cochrane Collaboration, London, UK) and Stata 12.0 (4905 Lakeway Drive, College Station, TX 77845 USA).

## Result

### Literature selection

**Fig. 1:**
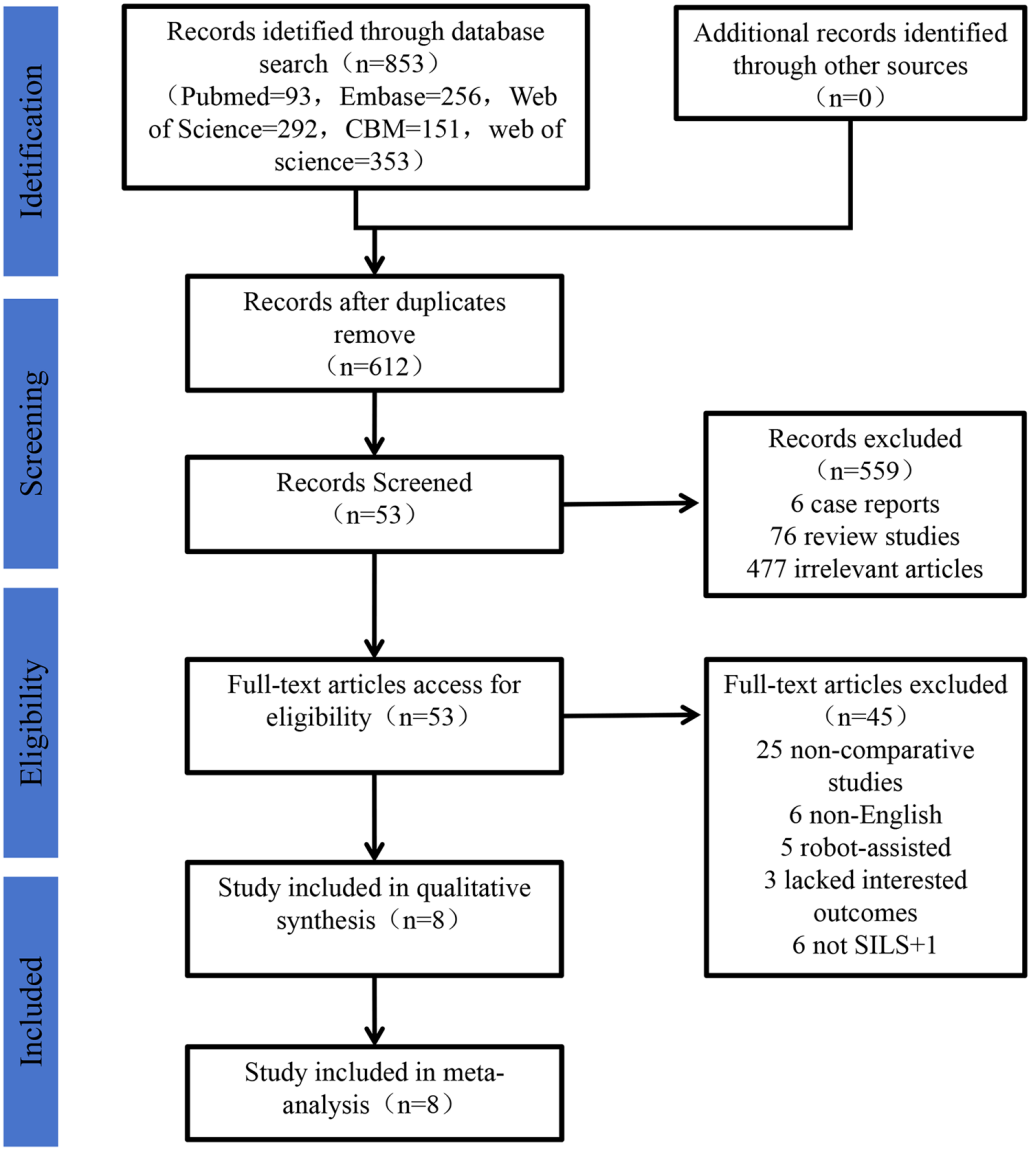
PRISMA flow diagram

The initial database search identified a total of 853 articles. After removing 241 duplicates, 612 articles remained. A thematic screening of the abstracts for these 612 articles was conducted, leading to the exclusion of 6 case reports, 76 review studies, and 477 articles unrelated to the topic. The remaining 53 articles underwent full-text screening. Following the application of inclusion and exclusion criteria, 25 were non-comparative studies, 6 were non-English publications, 6 did not provide surgical details, 5 involved robot-assisted procedures, and 3 lacked data on the outcomes of interest. Ultimately, 8 articles met the criteria for inclusion in this systematic review and meta-analysis (Fig. 1).

### Quality evaluation and basic characteristics

Randomized controlled trials and cohort studies underwent offset assessment and quality evaluation using the Cochrane risk-of-bias tool and NOS score scale, respectively. Seven high-quality studies, with a Cochrane score above 4 and an NOS score above 7, were included in this systematic review and meta-analysis (see Supplementary Fig. 1 and Supplementary Table 2).

Following a comprehensive quality assessment, the study conducted by Yu et al. was excluded from this meta-analysis. This decision was based on identified selection bias stemming from variations in patient sources, inadequate comparability due to baseline differences, and a score below 7 points. The final set of included studies comprised seven, involving one randomized controlled trial, three propensity score-matched studies, and three retrospective cohort studies. The investigations spanned from 2011 to 2022, including five studies conducted in China and two in Korea. Table 1 provides a comprehensive overview of the characteristics and demographic information of the patients across the eight studies included. A total of 1740 patients were allocated to treatment in these seven studies. Among these, 596 patients underwent SILS + 1 (34.2%), while 1144 patients underwent CLS (65.7%).

**Table 1:** Basic characteristics and quality assessment of the studies included in this meta-analysis

Study ID	Period	Design	No. of patient		Age, year		Male		BMI, kg/m^2^		ASA score		Tumor site	Study quality (NOS)
			SILS + 1	CLS	SILS + 1	CLS	SILS + 1	CLS	SILS + 1	CLS	SILS + 1	CLS		
Kang 2018 [24]	2011–2017	PSM	95	95	66.4 ± 9.7	67.5 ± 10.9	59	59	24.1 ± 3.1	24.1 ± 3.4	I/II/III 26/66/3	I/II/III 23/71/1	RC/RHC	7
Liu 2017 [25]	2011–2014	PSM	32	48	57.5 ± 10.8	55.5 ± 12.4	13	26	22.8 ± 2.3	22.3 ± 3.4	I + II/III 45/3	I + II/III 27/5	RC/SCC	9
Wu M 2023 [26]	2015–2018	PSM	258	516	58.4 ± 11.6	58.4 ± 21.3	156	325	22.1 ± 3.0	22.15 ± 2.8	I/II/III 5/190/14	I/II/III 117/373/26	CRC	9
Jiang 2023 [15]	2019–2022	RCS	25	56	62.0 ± 10.1	60.8 ± 12.7	15	36	23.1 ± 3.0	22.2 ± 2.5	I/II/III 3/17/5	I/II/III 6/40/10	URC/SCC	9
Song 2016 [27]	2011–2013	RCS	32	217	65.2 ± 10.8	64.6 ± 12.7	19	128	23.3 ± 2.8	23.9 ± 3.9	I/II/III 1/29/2	I/II/III 8/193/16	ACC/SCC	7
Wu H 2020 [28]	2015–2019	RCS	62	119	67.0 ± 2.5	66.0 ± 2.8	41	76	22 ± 1.5	21 ± 2.3	I/II/III 39/19/4	I/II/III 77/35/7	URC	8
Zhang 2023 [29]	2014–2016	RCT	92	93	56.9 ± 11.5	57.2 ± 11.7	49	56	22.8 ± 2.7	23.0 ± 3.1	I/II/III 53/34/5	I/II/III 53/35/5	RC/SCC	/
Yu 2016 [30]	2010–2013	PCS	38	92	65.7 ± 11.1	65.4 ± 10.4	26	45	23.6 ± 3.1	24.3 ± 3.2	I + II/III 33/5	I + II/III 76/16	CC	6

*PSM* propensity-score matched studies, *RCS* retrospective cohort study, *RCT* randomized controlled trial, *SILS* + *1* single-incision plus one-port laparoscopy surgery, *CLS* conventional laparoscopy surgery, *ASA* American Society of Anesthesiologists, *RC* rectal cancer, *RHC* right hemicolon, *SCC* sigmoid colon cancer, *CRC* colorectal cancer, *URC* upper rectal cancer, *ACC* ascending colon cancer, *CC* colon cancer, *NOS* Newcastle–Ottawa Scale

### Intraoperative outcomes

#### Operation time and blood loss

**Fig. 2:**
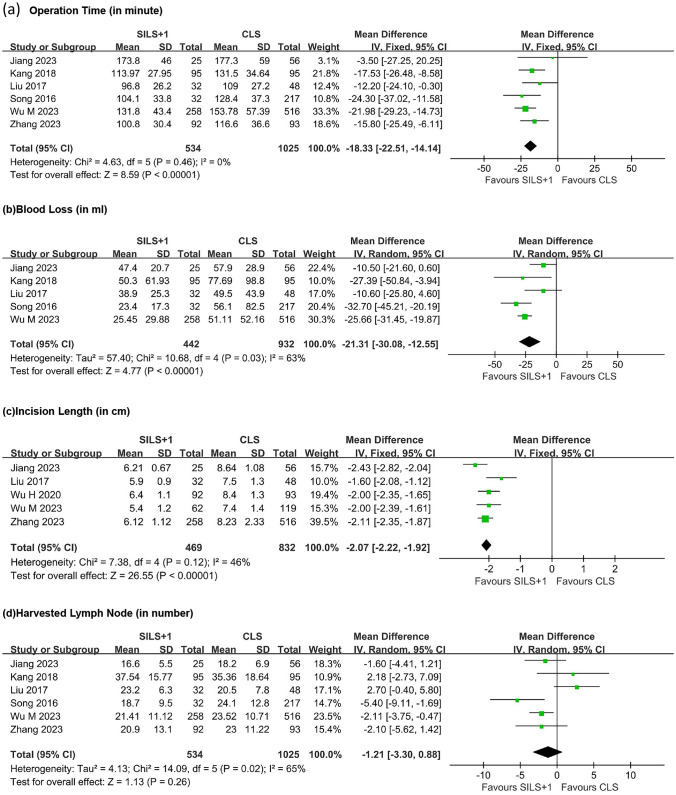
Forest plot comparing SILS + 1 with CLS. **a** Operation time. **b** Blood loss. **c** Incision length. **d** Harvested lymph node. SILS + 1, single-incision plus one-port laparoscopy surgery; CLS, conventional laparoscopy surgery; SD, standard deviation; CI, confidence interval

The analysis encompassing six studies investigated operation time (Fig. 2a), revealing no significant heterogeneity among groups based on the heterogeneity test (*τ*^2^ = 4.63, df = 5, *P* = 0.46, *I*^2^ = 0%). Consequently, a fixed-effect model was employed for combination. The results indicated a notable reduction in operation time in the SILS + 1 group compared to the CLS group (WMD =  − 18.33, 95% CI − 22.51 to − 14.14, *Z* = 8.59, *P* < 0.00001).

Five studies investigated intraoperative blood loss (Fig. 2b), revealing moderate heterogeneity among the groups (*τ*^2^ = 57.40, *Χ*^2^ = 10.68, df = 4, *P* = 0.03, *I*^2^ = 63%). A random-effects model was applied for data synthesis, indicating that the SILS + 1 group had significantly lower intraoperative blood loss compared to the CLS group (WMD =  − 21.31, 95% CI − 30.08 to − 12.55, *Z* = 5.10, *P* < 0.00001).

#### Incision length and harvested lymph node

Five studies investigated the incision length (Fig. 2c), showing mild heterogeneity among groups (*τ*^2^ = 7.38, df = 4, *P* = 0.12, *I*^2^ = 46%). The results indicated that the incision length in the SILS + 1 group was shorter than that in the CLS group (WMD =  − 2.07, 95% CI − 2.22 to − 1.92, *Z* = 26.55, *P* < 0.00001).

Six studies investigated the number of harvested lymph nodes during surgery (Fig. 2d), showing moderate heterogeneity among the groups (*τ*^2^ = 4.13, *Χ*^2^ = 14.09, df = 5, *P* = 0.02, *I*^2^ = 65%). The random-effects model was applied for data synthesis, suggesting no difference in the number of detected lymph nodes between the SILS + 1 group and the CLS group (WMD =  − 1.21, 95% CI − 3.30 to 0.88, *Z* = 1.08, *P* = 0.26).

#### Proximal and distal resection margins

**Fig. 3:**
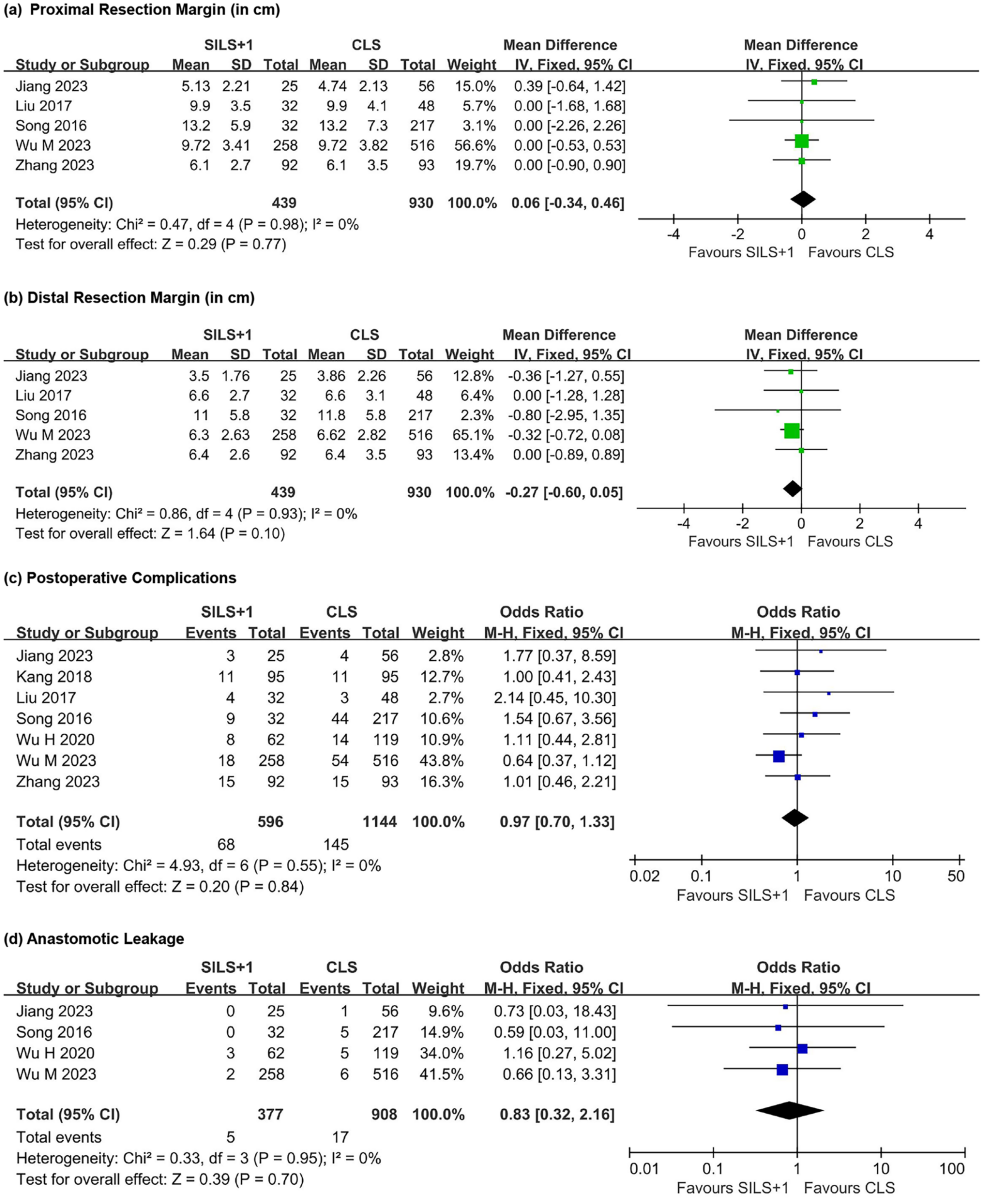
Forest plot comparing SILS + 1 with CLS. **a** Proximal resection margin. **b** Distal resection margin. **c** Postoperative complications. **d** Anastomotic leakage. SILS + 1, single-incision plus one-port laparoscopy surgery; CLS, conventional laparoscopy surgery; SD, standard deviation; ORs, odd ratios; CI, confidence interval

Five studies investigated the differences between the two groups regarding the proximal and distal margins. In terms of the proximal margin (Fig. 3a), there was no heterogeneity among the groups (*τ*^2^ = 0.47, df = 4, *P* = 0.98, *I*^2^ = 0%). Utilizing a fixed-effect model for data synthesis revealed no difference between the two groups (WMD = 0.06, 95% CI − 0.34 to 0.46, *Z* = 0.29, *P* = 0.77).

Concerning the distal margin (Fig. 3b), there was no heterogeneity among the groups (*τ*^2^ = 0.86, df = 4, *P* = 0.93, *I*^2^ = 0%). The analysis indicated that there was no statistically difference between the two groups concerning the distal margin (WMD =  − 0.27, 95% CI − 0.60 to 0.05, *Z* = 1.64, *P* = 0.10).

### Complications

#### Postoperative complications

All seven studies investigated postoperative complications (Fig. 3c). Heterogeneity analysis showed no heterogeneity among the groups (*τ*^2^ = 4.93, df = 6, *P* = 0.55, *I*^2^ = 0%). A fixed-effects model was employed for data synthesis, indicating no significant difference in postoperative complications between the SILS + 1 group (11.4%) and the CLS group (12.7%) (OR = 0.97, 95% CI 0.70 to 1.33, *Z* = 0.20, *P* = 0.84).

#### Anastomotic leakage

Four studies investigated the occurrence of anastomotic leakage (Fig. 3d), and there was no heterogeneity among the groups (*τ*^2^ = 0.64, df = 3, *P* = 0.89, *I*^2^ = 0%). The results indicated no significant difference in the incidence of anastomotic leakage between the SILS + 1 group (1.33%) and the CLS group (1.87%) (OR = 0.83, 95% CI 0.32 to 2.16, *Z* = 0.39, *P* = 0.70).

### Postoperative recovery

#### Time to first flatus and defecation

**Fig. 4:**
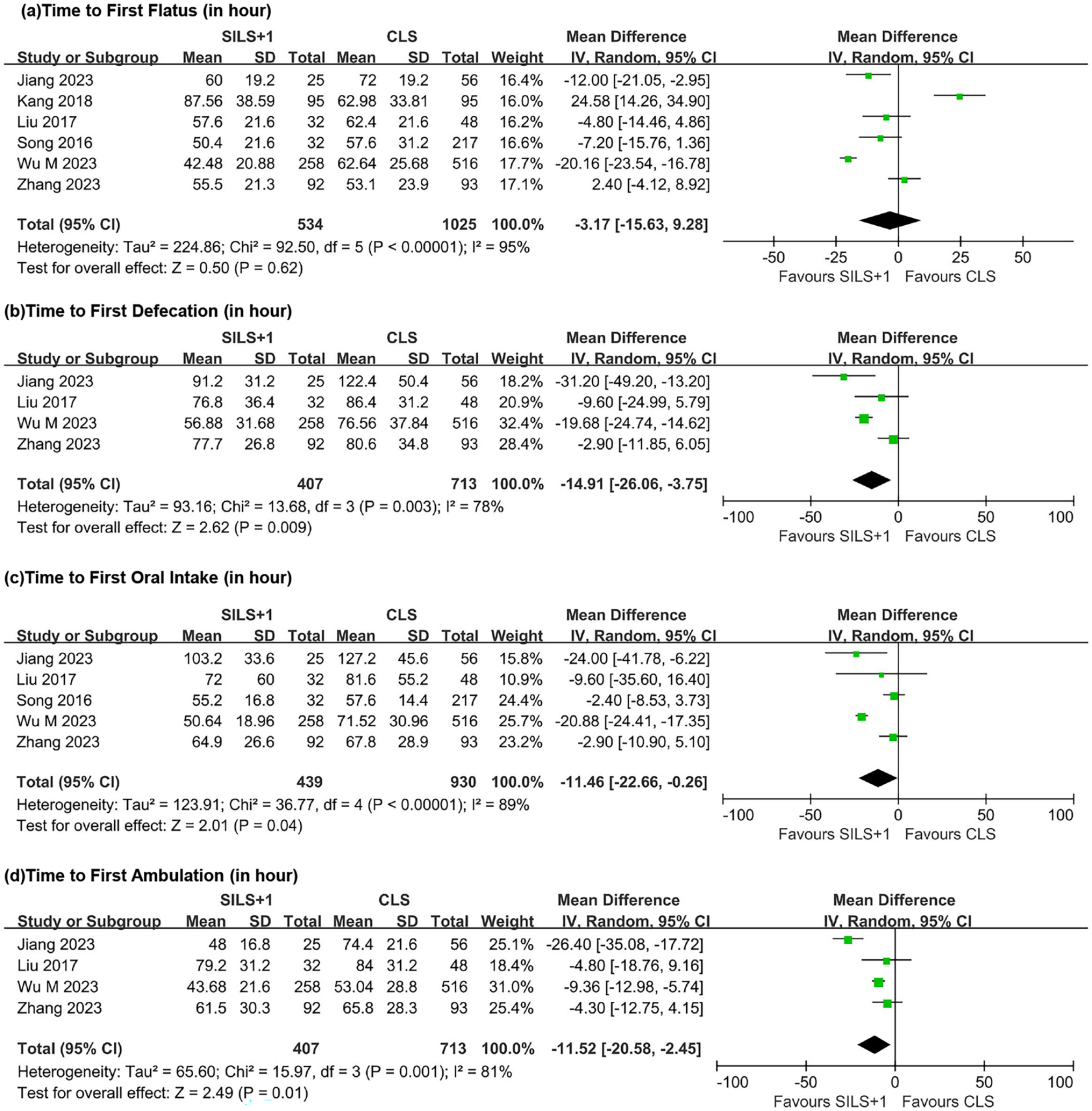
Forest plot comparing SILS + 1 with CLS. **a** Time to first flatus. **b** Time to first defecation. **c** Time to first oral intake. **d** Time to first ambulation. SILS + 1, single-incision plus one-port laparoscopy surgery; CLS, conventional laparoscopy surgery; SD, standard deviation; CI, confidence interval

Six studies investigated the time to first flatus (Fig. 4a), and significant heterogeneity was observed between groups (*τ*^2^ = 224.86, *Χ*^2^ = 92.50, df = 5, *P* < 0.00001, *I*^2^ = 95%). After analyzing with a random-effects model, the results indicated no statistically significant difference in the time to the first flatus between the two groups (WMD =  − 3.17, 95% CI − 15.63 to 9.28, *Z* = 0.50, *P* = 0.62).

Four studies investigated the time to the first postoperative defecation (Fig. 4b), and significant heterogeneity was observed between groups (*τ*^2^ = 93.16, *Χ*^2^ = 13.68, df = 3, *P* = 0.003, *I*^2^ = 78%). A random-effects model was used. The SILS + 1 group, compared to the CLS group, had a shorter time to the first postoperative bowel movement (WMD =  − 14.91, 95% CI − 26.06 to − 3.75, *Z* = 2.62, *P* = 0.009).

#### Time to first oral intake and ambulation

Five studies investigated the time of first postoperative oral intake (Fig. 4c) with large heterogeneity between groups (*τ*^2^ = 123.91, *Χ*^2^ = 36.77, df = 4, *P* < 0.00001, *I*^2^ = 89%) using a random effects model. The SILS + 1 group was shorter compared with the CLS group, but did not achieve statistical efficacy (WMD =  − 11.46, 95% CI − 22.66 to − 0.26, *Z* = 2.01, *P* = 0.04).

Four studies investigated the time to first ambulation (Fig. 4d), and there was significant heterogeneity between groups (*τ*^2^ = 65.60, *Χ*^2^ = 15.97, df = 3, *P* = 0.001, *I*^2^ = 81%). A random-effects model was used for data synthesis. SILS + 1 group had a shorter time to ambulation (WMD =  − 11.52, 95% CI − 20.58 to − 2.45, *Z* = 2.49, *P* = 0.01).

#### Hospital stay

**Fig. 5:**
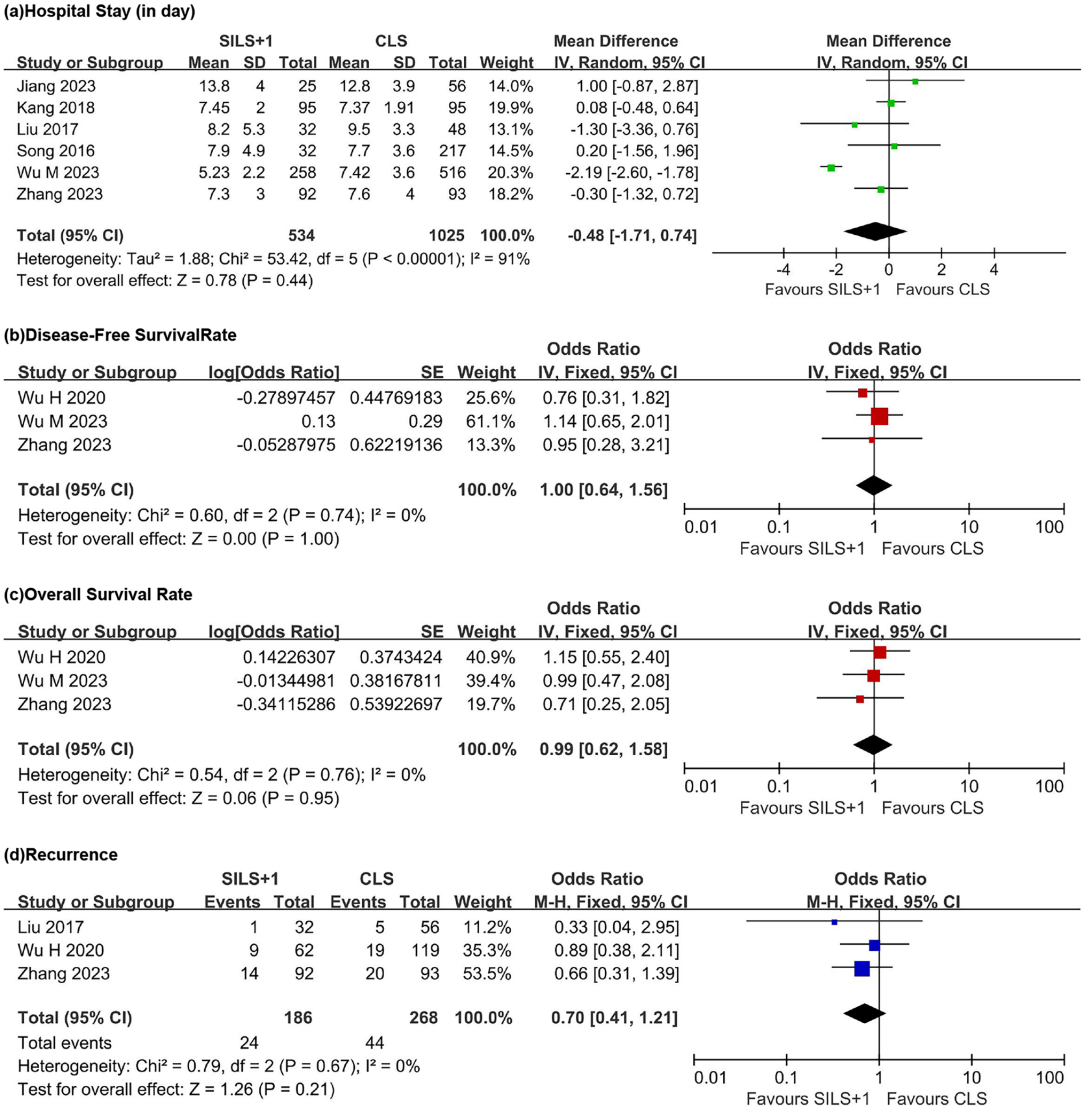
Forest plot comparing SILS + 1 with CLS. **a** Hospital stay. **b** Disease-free survival rate. **c** Overall survival rate. **d** Recurrence. SILS + 1, single-incision plus one-port laparoscopy surgery; CLS, conventional laparoscopy surgery; SD, standard deviation; CI, confidence interval

Six studies investigated the length of hospital stay (Fig. 5a), and there was significant heterogeneity between groups (*τ*^2^ = 1.88, *Χ*^2^ = 53.42, df = 5, *P* < 0.00001, *I*^2^ = 91%). The random effects model suggested no difference between the two groups (WMD =  − 0.48, 95% CI − 1.71 to 0.74, *Z* = 0.78, *P* = 0.44).

### Prognosis

#### Disease-free survival rate and overall survival rate

Three studies investigated the disease-free survival rate (Fig. 5b) of the two groups, and there was no heterogeneity between the groups (*τ*^2^ = 0.60, df = 2, *P* = 0.74, *I*^2^ = 0%). Using a fixed-effects model for data synthesis, the results suggested that there was no difference in overall survival rate between the SILS + 1 group and the CLS group (OR = 1.00, 95% CI 0.64 to 1.56, *Z* = 0.00, *P* = 1.00).

Three studies investigated the overall survival rate (Fig. 5c) of the two groups, and there was no heterogeneity between the groups (*τ*^2^ = 0.54, df = 2, *P* = 0.76, *I*^2^ = 0%). The synthesis of data using a fixed-effects model suggested that there was no difference in overall survival rate between the SILS + 1 group and the CLS group (OR = 0.99, 95% CI 0.62 to 1.58, *Z* = 0.06, *P* = 0.95).

#### Recurrence

Three studies investigated tumor recurrence (Fig. 5d) after surgery in the two groups, and there was no heterogeneity between the groups (*τ*^2^ = 079, df = 2, *P* = 0.67, *I*^2^ = 0%). The synthesis of data using a fixed-effects model suggested that there was no difference in tumor recurrence after surgery between the SILS + 1 group (1.29%) and the CLS group (1.69%) (OR = 0.70, 95% CI 0.41 to 1.21, *Z* = 1.26, *P* = 0.21).

### Subgroup analysis

**Fig. 6:**
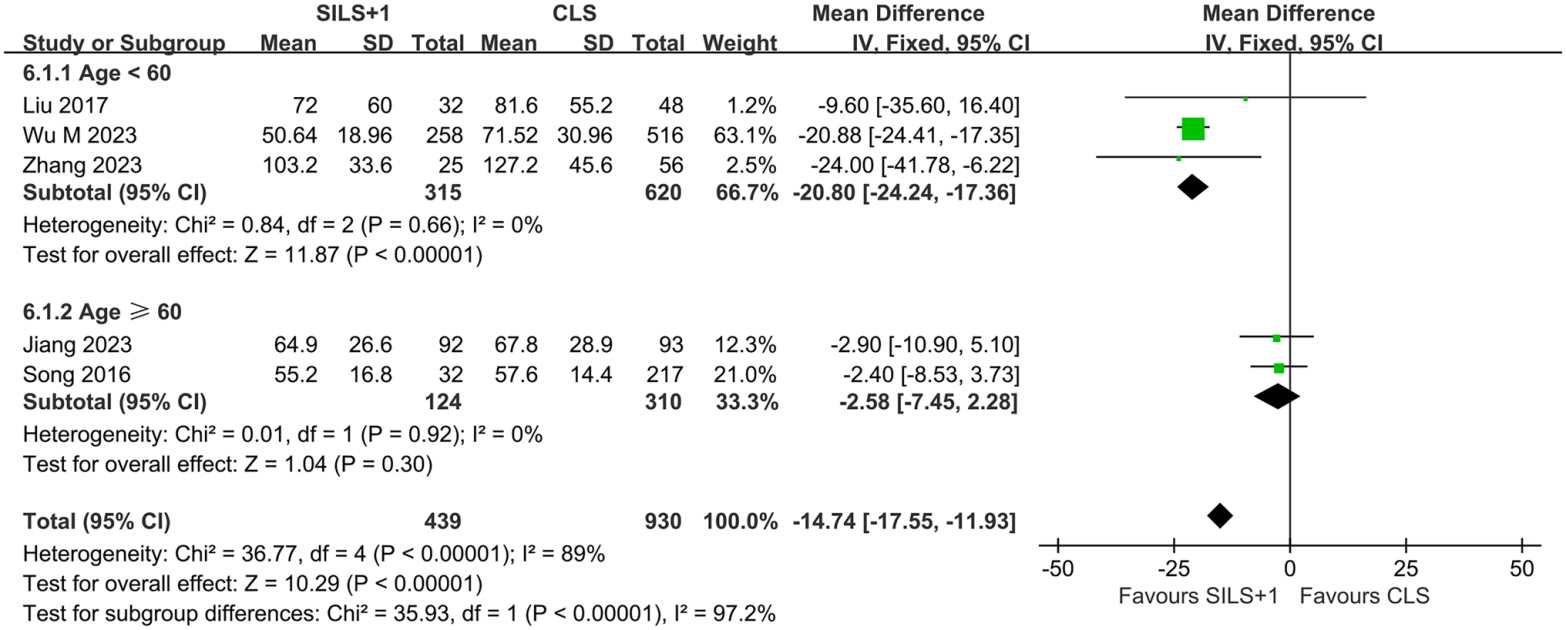
Forest plot of subgroup analysis for first oral intake time. SILS + 1, single-incision plus one-port laparoscopy surgery; CLS, conventional laparoscopy surgery; SD, standard deviation; CI, confidence interval

Subgroup analysis was conducted based on age, sample size, tumor site, tumor size, and operation time for time to postoperative first oral intake. The results revealed that when grouped by age, both subgroups exhibited zero heterogeneity, indicating that age is the main source of heterogeneity for postoperative eating time (Fig. 6). In the subgroup with age less than 60 years, the SILS + 1 group demonstrated a shorter postoperative eating time compared to the CLS group (WMD =  − 20.80, 95% CI − 24.24 to − 17.36, *Z* = 11.87, *P* < 0.00001). However, in the elderly subgroup (≥ 60 years), there was no statistically significant difference in postoperative eating time between the SILS + 1 group and the CLS group (WMD =  − 2.58, 95% CI − 7.45 to 2.28, *Z* = 1.04, *P* = 0.30). No heterogeneity source was found in the analysis of other subgroups (see Supplementary Fig. 2).

### Heterogeneity and sensitivity analysis

In-depth exploration of substantial heterogeneity was meticulously conducted through a “leave-one-out” analysis, systematically scrutinizing each study’s exclusion to pinpoint the origins of the observed heterogeneity.

Concerning the first defecation time, the study by Zhang et al. was identified as the primary source of heterogeneity. After excluding the results of this study, the overall results demonstrated lower heterogeneity (*τ*^2^ = 24.62, *Χ*^2^ = 3.22, df = 2, *P* = 0.20; *I*^2^ = 38%). The analysis using a fixed-effects model continued to support the aforementioned results (WMD =  − 19.53, 95% CI − 24.17 to − 14.89, *Z* = 8.24, *P* < 0.00001) (see Supplementary Fig. 3a). However, upon thorough review of the included studies, a reasonable explanation for the source of heterogeneity could not be identified. This underscores the need for caution in the interpretation of results and suggests the potential for inherent variability in the data that may not be readily elucidated.

Upon analyzing the results for the first ambulation time, the study by Jiang et al. emerged as the primary source of heterogeneity. After the exclusion of this study, the heterogeneity test results indicated (*τ*^2^ = 0.00, *Χ*^2^ = 1.43, df = 2, *P* = 0.49, *I*^2^ = 0%), and the results from the fixed-effects model analysis continued to support the above conclusion (WMD =  − 8.37, 95% CI − 11.61 to − 5.13, *Z* = 5.07, *P* < 0.00001) (see Supplementary Fig. 3b). It is noteworthy that the mean age of the subjects in Jiang et al.’s study was over 60, while the mean age in the other studies was less than 60 years old. This divergence in age demographics suggests that the delayed postoperative ambulation in elderly patients may be a potential source of heterogeneity. This observation emphasizes the importance of considering demographic characteristics when interpreting results and highlights the impact of patient age on postoperative outcomes.

The analysis of hospital stay revealed that the study by Wu M et al. was the origin of heterogeneity. Upon the exclusion of the study by Wu M et al., heterogeneity diminished to zero (*τ*^2^ = 0.00, *Χ*^2^ = 3.09, df = 4, *P* = 0.54, *I*^2^ = 0%). Employing a fixed-effects model for data synthesis after the exclusion, no statistically significant difference in postoperative hospital stay between the two groups was observed (WMD = 0.00, 95% CI − 0.44 to 0.45, *Z* = 0.01, *P* = 0.99) (see Supplementary Fig. 3c). Notably, among the six studies included, patients in the other five underwent surgery from the rectum to the sigmoid colon, while Wu M et al.’s study involved surgeries in both the ascending and descending colon. This variance in tumor location emerges as a plausible source of heterogeneity, underscoring the impact of considering specific procedural details in interpreting outcomes.

Upon conducting a sensitivity analysis for all outcome indicators, the robustness of the overall results was evident for several parameters, including operation time, blood loss, incision length, postoperative complications, anastomotic leakage, first defecation time, recurrence, hospital stay, proximal margin, and distal margin. The removal of any single study did not sway these results (see Supplementary Figs. 4–5).

**Fig. 7:**
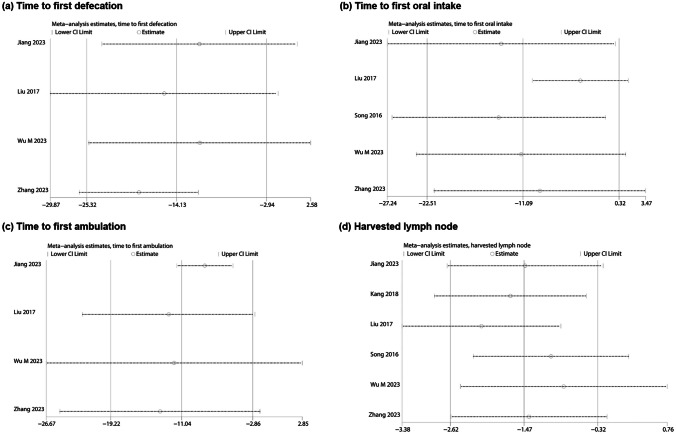
Sensitivity analysis of unstable results. **a** Time to first defecation. **b** Time to first oral intake. **c** Time to first ambulation. **d** Harvested lymph node. CI, confidence interval

However, a nuanced pattern emerged for first defecation time, first activity time, first oral intake time, and lymph node retrieval. In these domains, the sensitivity analysis revealed a degree of instability and a decrease in the quality of evidence, emphasizing the need for cautious interpretation and consideration of the specific studies contributing to these outcomes (Fig. 7).

### Publication bias

The presence of publication bias was assessed using Harbord’s test for binary variable [31] and Egger’s test for continuous variable. The results indicated that there was no significant publication bias for all outcome measures (all *P* values > 0.05) (Table 2).

**Table 2:** Quantitative assessment of publication bias for outcomes

Outcomes	Data type	Egger/Harbord	Publication bias
Operation time	Continuous variable	0.23	None
Blood loss	Continuous variable	0.541	None
Incision length	Continuous variable	0.746	None
Harvested lymph node	Continuous variable	0.639	None
Proximal resection margin	Continuous variable	0.579	None
Distal resection margin	Continuous variable	0.869	None
Postoperative complications	Binary variable	0.104	None
Anastomotic leakage	Binary variable	0.093	None
Time to first flatus	Continuous variable	0.082	None
Time to first defecation	Continuous variable	0.797	None
Time to first oral intake	Continuous variable	0.527	None
Time to first ambulation	Continuous variable	0.782	None
Hospital stay	Continuous variable	0.31	None
Recurrence	Binary variable	0.479	None
DFS	Continuous variable	0.538	None
OS	Continuous variable	0.226	None

Egger/Harbord’s *P* value > 0.05 indicates the absence of publication bias

*DFS* disease-free survival rate, *OS* overall survival rate

## Discussion

In the past few decades, laparoscopic surgery has gradually replaced open surgery as the preferred choice for colorectal cancer surgery due to its minimal invasiveness and oncological efficacy [1–3]. With advancements in surgical instruments and surgeons’ skills, minimally invasive surgery has become the current goal of surgical practice. Single-incision laparoscopic surgery (SILS) is considered a hallmark of progress in minimally invasive surgery, and since its first application in colorectal cancer by Bucher et al. [32] and Remzi et al. [33] in 2008, it has received extensive research attention. The latest meta-analysis results indicate that SILS is superior to conventional laparoscopic surgery (CLS) in terms of incision length, lymph node retrieval, overall complication rates, time to bowel movement, time to flatus, pain scores, and length of hospital stay in colorectal cancer patients [34]. Additionally, SILS reduces the potential risks associated with trocar-related complications, such as small bowel injury, vascular injury during trocar insertion, port site herniation, and recurrence [35]. However, SILS has not been widely adopted mainly due to the high technical challenges it poses to surgeons, including loss of triangulation, instrument collisions, suboptimal surgical exposure, and real-time visualization [12–14], as well as the risks of vascular and tissue damage during surgery [36]. Moreover, in distal sigmoid or rectal cancer, it is challenging to use a linear stapler in SILS, which may increase the risk of anastomotic leakage or inadequate distal margin [37]. Therefore, to overcome these challenges, additional ports are introduced into SILS, referred to as SILS + 1.

Based on SILS, SILS + 1 typically adds a 12-mm trocar needle in the right or left lower abdomen depending on the surgical site. This additional port restores the triangulation of laparoscopic surgery, thereby reducing instrument collisions, improving visibility, and tissue tension [38]. Additionally, it can serve as an entry point for linear staplers and a channel for drainage tubes [35]. Adair et al. [39] reported that adding another port in SILS is a more practical technique used in clinical settings. Results from Wu et al.’s propensity score-matched study support that SILS + 1 is as safe, feasible, and oncologically effective as CLS for treating colorectal cancer [26]. Moreover, SILS + 1 offers advantages such as reduced surgical time, decreased blood loss, better cosmetic outcomes, less pain, and faster recovery [26]. In a recent randomized controlled trial assessing the long-term efficacy of SILS + 1, the SILS + 1 group had a median follow-up time of 64.0 months, while the CLS group had 65.0 months. SILS + 1 demonstrated similar 3-year disease-free survival (DFS) (87.8% vs. 86.9%) and 5-year overall survival (OS) (86.7% vs. 80.5%) compared to CLS [29]. Kim et al. also reported no significant differences in 3-year OS and DFS between the two groups (94.5 vs. 97.1%, 89.5 vs. 87.4%), with a median follow-up time of 29.5 months [10]. Furthermore, SILS + 1 exhibited the lowest postoperative inflammatory response compared to CLS and SILS, including WBC, CRP, IL-6, and TNF-α [25, 35]. However, current studies suffer from small sample sizes, insufficient evidence quality, and contradictory findings regarding surgical time, intraoperative blood loss, and lymph node retrieval [15, 25, 27]. Therefore, we conducted this study to provide stronger evidence supporting the safety and feasibility of SILS + 1. To our knowledge, this is the first meta-analysis and systematic review comparing SILS + 1 with CLS.

This study collected results from 7 randomized controlled trials and propensity score-matched studies comparing SILS + 1 and CLS, involving a total of 1740 patients with colorectal cancer. Among them, 596 patients received SILS + 1 treatment (34.2%), while 1144 patients received CLS treatment (65.7%). The cases included in these studies had comparable baseline characteristics. For instance, in the study by Wu et al., cases with a BMI > 28.0 kg/m^2^ and those not meeting the inclusion criteria were excluded [26]. Jiang et al.’s study included a BMI < 30.0 kg/m^2^ in their inclusion criteria [15]. This is because obesity is an independent risk factor for postoperative complications following laparoscopic right hemicolectomy [40]. Additionally, obesity (BMI > 30.0 kg/m^2^) is associated with increased cardiovascular burden, respiratory complications, as well as higher rates of conversion to open surgery and postoperative complications [41].

Our preliminary research results indicate that compared to CLS, SILS + 1 demonstrates advantages in various aspects including surgical time (WMD =  − 18.33, *P* < 0.00001), intraoperative blood loss (WMD =  − 21.31, *P* < 0.00001), incision length (WMD =  − 2.07, *P* < 0.00001), time to first defecation (WMD =  − 14.91, *P* = 0.009), time to first oral intake (WMD =  − 11.46, *P* = 0.04), and time to postoperative ambulation (WMD =  − 11.52, *P* = 0.01). However, there were no statistically significant differences between SILS + 1 and CLS in terms of lymph node retrieval, proximal and distal margins, complications, anastomotic leakage rate, length of hospital stay, disease-free survival, overall survival, and postoperative recurrence rate. Heterogeneity tests revealed high heterogeneity for intraoperative blood loss (*I*^2^ = 63%), lymph node retrieval (*I*^2^ = 65%), time to first flatus (*I*^2^ = 95%), time to first defecation (*I*^2^ = 78%), time to first oral intake (*I*^2^ = 89%), time to postoperative ambulation (*I*^2^ = 81%), and length of hospital stay (*I*^2^ = 91%). Due to limitations in the included study data, we only conducted subgroup analyses for time to first oral intake based on age, surgical time, tumor location, tumor size, and sample size. When grouped by whether the age exceeded 60 years in the included studies, heterogeneity disappeared within the two subgroups. This suggests that the difference in age among the study populations is the main source of heterogeneity in time to first oral intake. Additionally, compared to CLS, SILS + 1 exhibited a shorter time to first oral intake in the subgroup of patients under 60 years old (*P* < 0.00001), while there was no difference in the subgroup aged 60 years or older (*P* = 0.30). This indicates that SILS + 1 may only benefit younger patients (< 60 years old) in terms of postoperative oral intake time, which may be related to slower gastrointestinal function recovery in elderly patients [42]. However, this needs further support from additional research. Since subgroup analysis reduces the sample size within each group, it increases the possibility of statistical bias and lowers the level of evidence.

A “leave-one-out” analysis of the high heterogeneity results suggests that age and tumor location may be sources of high heterogeneity in time to first ambulation and length of hospital stay. In colorectal cancer patients, advanced age is an independent risk factor for postoperative complications, which affects postoperative recovery [40]. Among all included studies, there was no objectively unified discharge standard provided, and there may be a possibility of physicians adopting approximate postoperative observation times for patients with different recovery levels [43]. This inevitably introduces potential heterogeneity. Sensitivity analysis of all outcome indicators showed instability only in the time to first defecation, time to first ambulation, time to oral intake, and the number of lymph nodes harvested. This instability reduced the quality grade, indicating the need for further research to confirm these findings. Additionally, the Harbord test for categorical variables and the Egger test for continuous variables were used to assess the presence of publication bias. The results demonstrated that there was no significant publication bias across all outcome measures.

Surgical duration is a critical parameter that signifies the feasibility of the surgical process [44]. There has been ongoing debate regarding the differences in surgery duration between SILS + 1 and CLS. In most studies, SILS + 1 consistently demonstrated shorter surgery durations compared to CLS [15, 25, 27, 45]. Studies by Song et al. [27] and Yu et al. [30] suggested that the shorter surgery duration of SILS + 1 compared to CLS was attributed to the presence of selection bias. In their studies, the SILS + 1 group had earlier tumor staging, smaller tumor volumes, and more skilled surgeons. In laparoscopic surgery, for every 1% increase in team familiarity, surgery duration decreases by approximately 0.24% [46]. Single-port laparoscopy often requires more experienced surgeons, and a higher level of expertise and experience may significantly reduce surgery duration [47, 48], which also applies to single-port plus one laparoscopic surgery. However, in further propensity score matching experiments, surgeries in both groups were performed by a single surgeon, and the operating time for single-port surgeries was shorter than that for multi-port surgeries (114.4 ± 28.7 min vs. 126.7 ± 34.5 min, *P* = 0.021) [24]. However, the choice of surgical approach for patients is determined by the attending physician, introducing selection bias. Many scholars believe that the shorter incision length in the SILS + 1 group compared to the CLS group is the main reason for the shorter surgery duration [24, 49]. Closing the abdominal wall incision is typically performed by junior physicians, which may magnify the impact of incision length on surgery duration [24]. SILS + 1 utilizes the original incision to extract specimens. Although the original incision in SILS + 1 may also need to be extended, it still requires less time than CLS due to its initial longer length [49]. Zhang et al.’s [29] randomized controlled trial evaluated the qualifications of surgeons to reduce the impact of surgical experience on surgery duration, and the results showed that the total surgery time in the SILS + 1 group was significantly shorter than that in the CLS group (100.8 ± 30.4 vs. 116.6 ± 36.6, *P* = 0.002). This reduction in time was mainly due to intra-abdominal surgery time (66.2 ± 26.9 vs. 76.3 ± 28.2, *P* = 0.014), rather than closure time (16.8 ± 5.8 vs. 18.7 ± 7.8, *P* = 0.067). The reduction in intra-abdominal surgery time in SILS + 1 may be due to the lack of coordination of inexperienced assistants in CLS [29]. Studies have confirmed that the familiarity among members of the laparoscopic team affects surgery duration, and the lack of coordination among surgical assistants may even cause unnecessary interruptions during surgery [46]. Therefore, compared to CLS, under appropriate conditions, the role of surgical assistants can be replaced by the left hand of an experienced surgeon, or even reduce surgery time. In SILS + 1 surgery, surgeons operate independently without assistants, avoiding the time required for adjustment and cooperation [45]. This evidence supports that, compared to CLS, SILS + 1 does not significantly increase surgical difficulty, and it leads to faster postoperative recovery while maintaining similar safety, feasibility, and oncological efficacy.

This study has several acknowledged limitations. Although the Cochrane Handbook recommends combining randomized controlled trials and non-randomized controlled trials in meta-analyses, we included various types of studies due to limitations in the current literature quantity. This introduced inherent flaws associated with selection bias, which could lead to uneven distribution of confounding factors. Some results in this meta-analysis showed instability in sensitivity analyses, reducing the quality of certain evidence. The existing data in current studies are insufficient to support comprehensive subgroup analyses, making the sources of heterogeneity in some conclusions uncertain. Therefore, larger, multicenter, prospective, randomized controlled trials, along with international prospective registration, are needed to provide more reliable evidence.

## Conclusion

The current evidence supports that SILS + 1 is equally safe, feasible, and effective in treating colorectal cancer compared to CLS. Compared to CLS, SILS + 1 demonstrates superiority in shortening the surgical incision and promoting postoperative recovery. SILS + 1 can provide a safe and feasible alternative to CLS.

## Supplementary Information

Below is the link to the electronic supplementary material.

Supplementary file1 (DOCX 27 KB)

Supplementary file2 (TIF 899 KB)

Supplementary file3 (TIF 4323 KB)

Supplementary file4 (TIF 1991 KB)

Supplementary file5 (TIF 1326 KB)

Supplementary file6 (TIF 357 KB)

## Data Availability

The datasets used and analyzed during the current study are available from the corresponding author upon reasonable request.
