# Supplementary figures and images for: Single-incision plus one-port laparoscopy surgery versus conventional multi-port laparoscopy surgery for colorectal cancer: a systematic review and meta-analysis
Source: Int J Colorectal Dis. 2024 Apr 29;39(1):62. doi: 10.1007/s00384-024-04630-x (PMC11058787; doi:10.1007/s00384-024-04630-x)

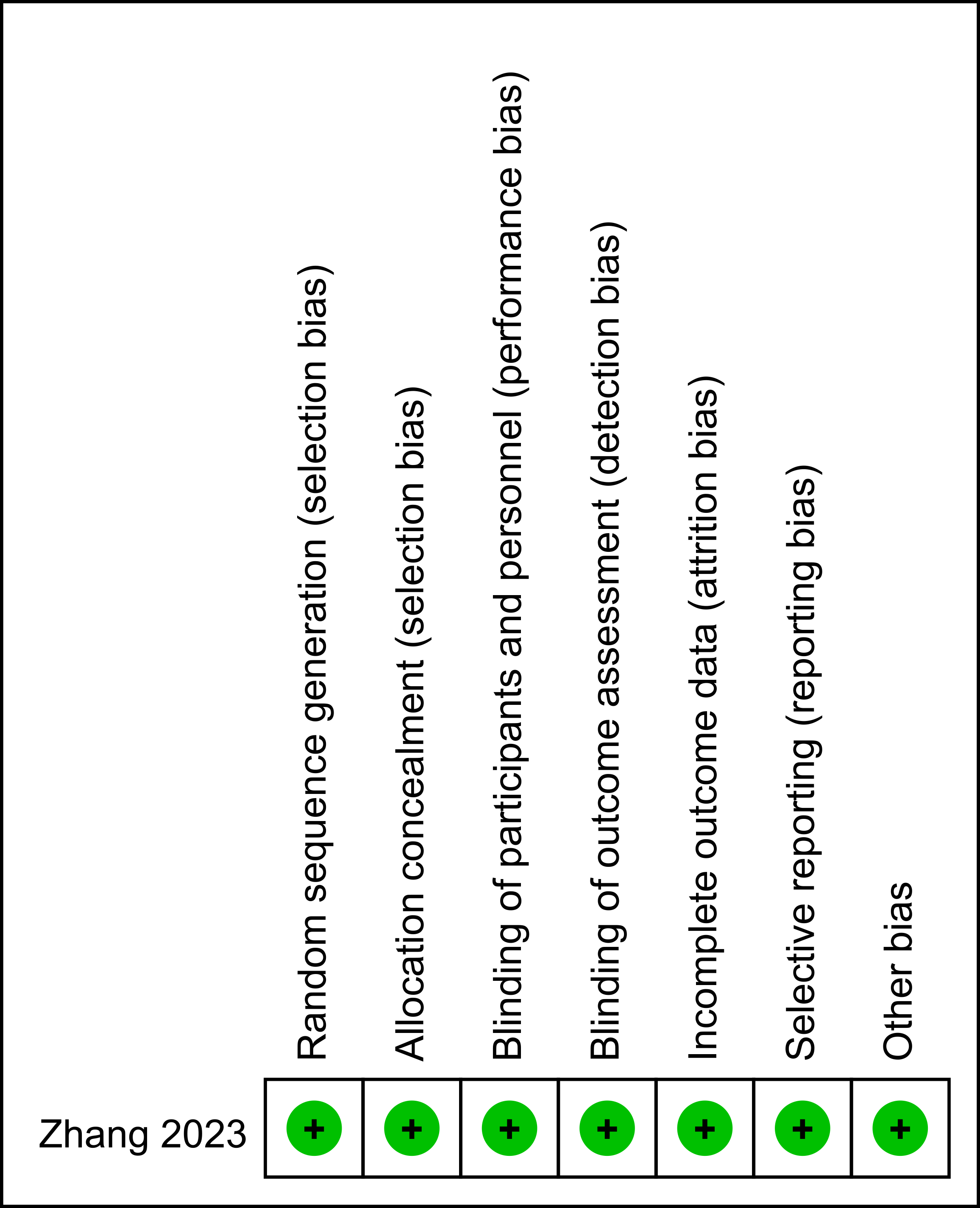

Supplement: Supplementary file 2 — Supplementary file2 (TIF 899 KB) [file 384_2024_4630_MOESM2_ESM.tif]

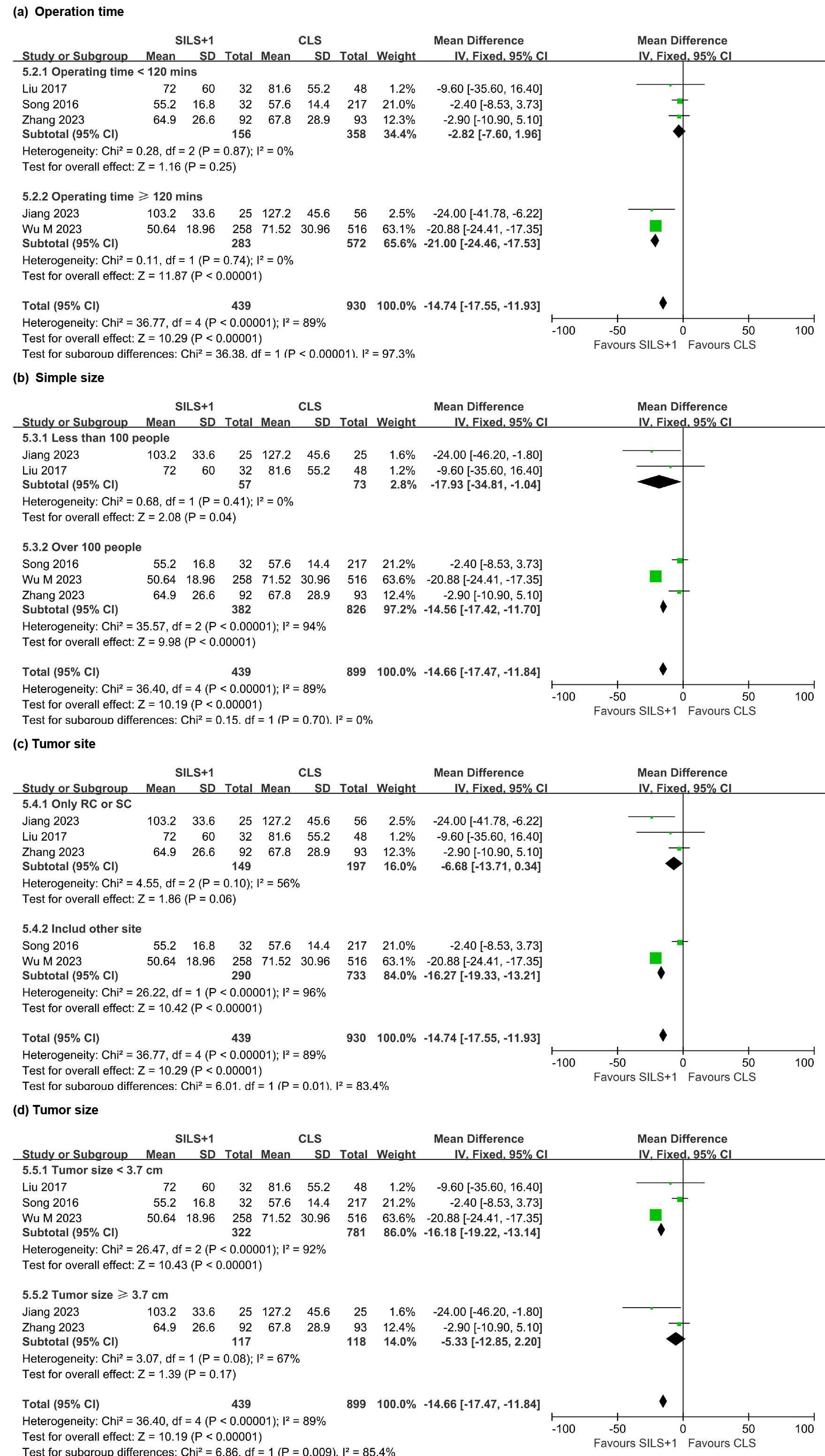

Supplement: Supplementary file 3 — Supplementary file3 (TIF 4323 KB) [file 384_2024_4630_MOESM3_ESM.tif]

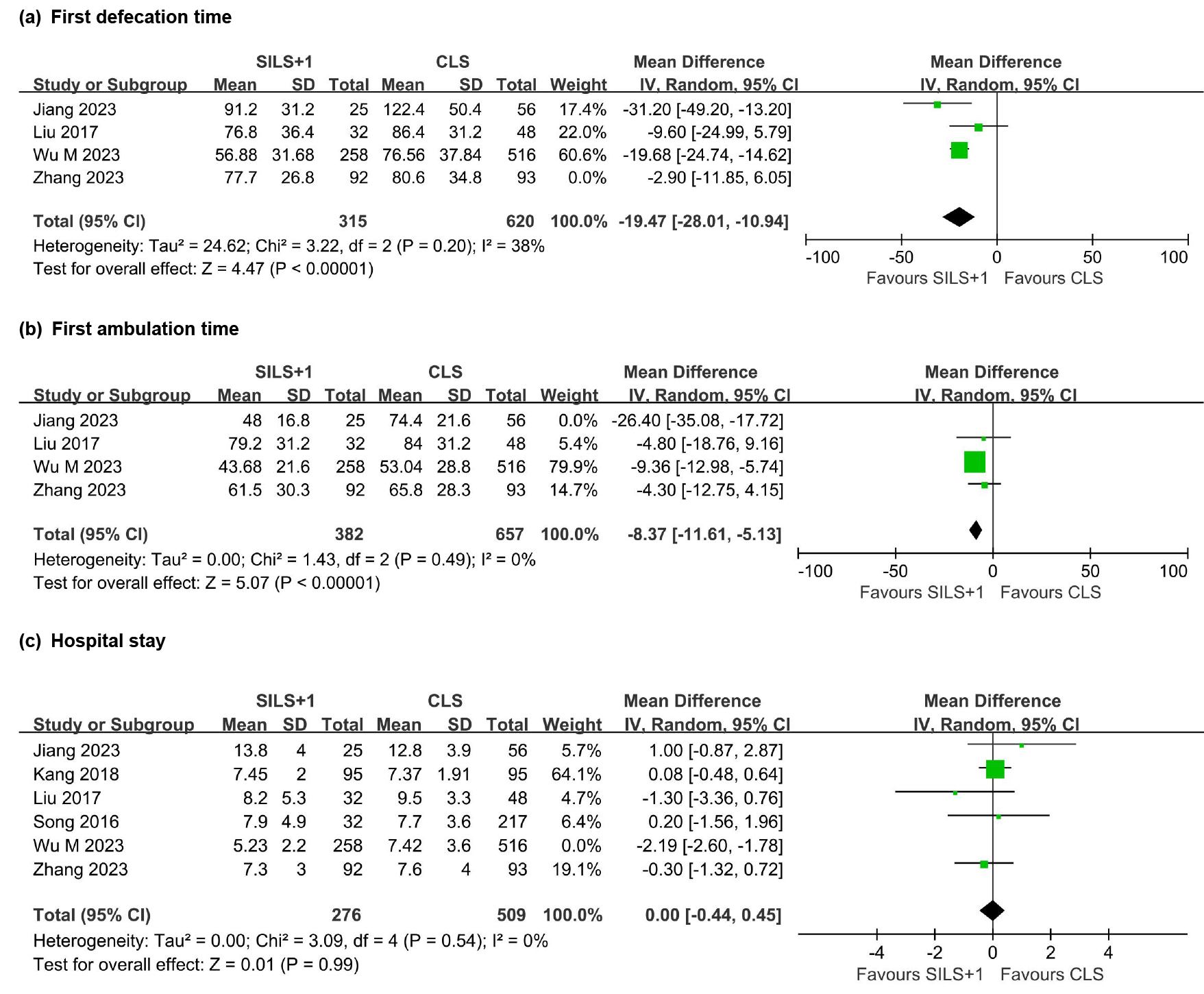

Supplement: Supplementary file 4 — Supplementary file4 (TIF 1991 KB) [file 384_2024_4630_MOESM4_ESM.tif]

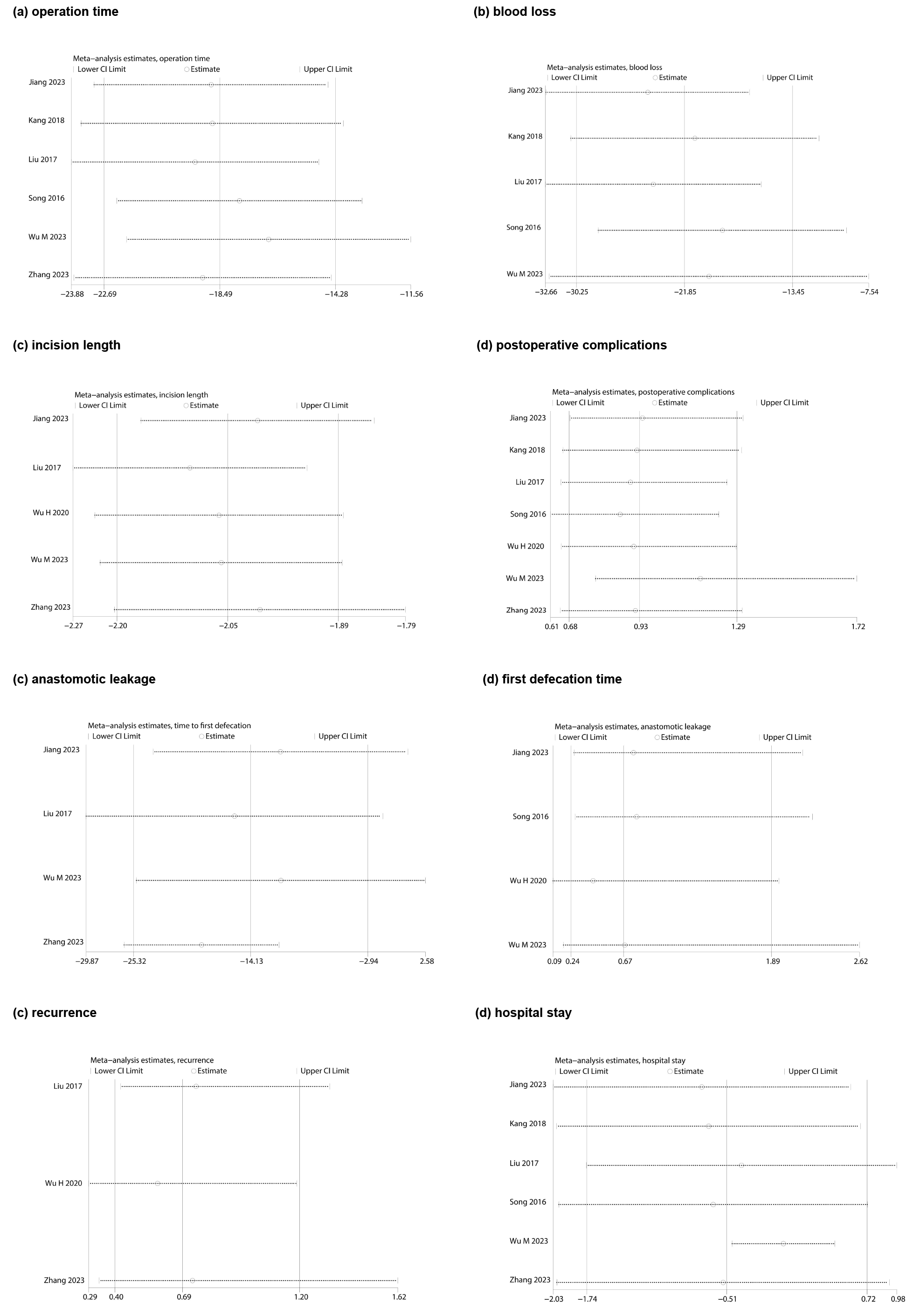

Supplement: Supplementary file 5 — Supplementary file5 (TIF 1326 KB) [file 384_2024_4630_MOESM5_ESM.tif]

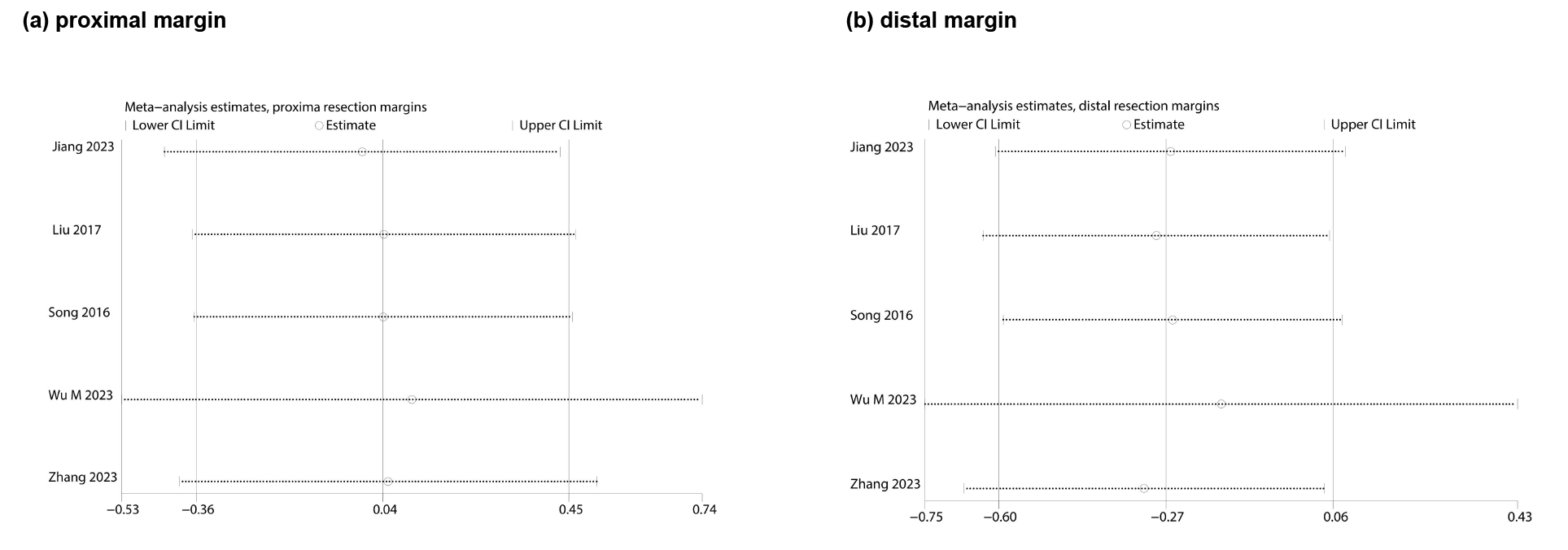

Supplement: Supplementary file 6 — Supplementary file6 (TIF 357 KB) [file 384_2024_4630_MOESM6_ESM.tif]
